# Lateral Lumbar Spinal Stenosis: Associations With the Oswestry Disability Index, Visual Analogue Scale, and Magnetic Resonance Imaging

**DOI:** 10.7759/cureus.50475

**Published:** 2023-12-13

**Authors:** Yusoff Norisyam, Azizul A Salim, Zairul Bahrin, Mohd I Yusof, Mohammad Paiman, Chandran Nadarajan

**Affiliations:** 1 Department of Orthopedics, Hospital Pulau Pinang, Georgetown, MYS; 2 Department of Orthopedics, Universiti Sains Malaysia, Kubang Kerian, MYS; 3 Department of Orthopedics, Hospital Universiti Sains Malaysia, Kota Bharu, MYS; 4 Department of Radiology, Hospital Universiti Sains Malaysia, Kota Bharu, MYS

**Keywords:** magnetic resonance imaging (mri), clinical features, visual analogue score, oswestry disability index, lateral spinal stenosis

## Abstract

Introduction

Degenerative lumbar spinal stenosis is a communal problem in the sixth decade of life involving L4/L5 and L5/S1 levels. Lateral spinal stenosis is often underestimated because of no established relationship between the clinical symptoms and MRI findings. We conducted a study to establish an association between the degree of anatomical lateral stenosis, posterior disc height, and disc degeneration from MRI with the daily disability and pain severity for lateral lumbar spinal stenosis.

Methods

This was a cross-sectional study involving 121 patients with distinct clinical symptoms of lateral lumbar spinal stenosis evaluated from February 2018 to December 2019. The clinical data were evaluated using the Oswestry Disability Index (ODI) and Visual Analogue Scale (VAS), while magnetic resonance imaging (MRI) was assessed qualitatively for the anatomical gradation of lateral spinal stenosis, the magnitude of posterior disc height, and the extent of disc degeneration. Statistical analysis for the correlation between posterior disc height and ODI and VAS scores was evaluated using Pearson's correlation test via SPSS version 23.0 (IBM Inc., Armonk, New York), and the association between the extent of lateral stenosis and disc degeneration on MRI with ODI and VAS scores was determined by the Fisher Exact Test via STATA version 14.0 (StataCorp LLC, College Station, Texas). The association was considered statistically significant with a P-value of less than 0.05.

Results

The analysis of 121 patients showed the mean age of the patients was 58.7 ± 7.1 years old. The number of female patients was higher compared to male patients, 52.9% and 47.1%, respectively. 97.5% of the patients were married or cohabiting, and 76.0% had an abnormal body mass index. The mean score of ODI and VAS was 62.2 ± 10.7% and 79.3 ± 8.6 respectively. 49.6% of the patient presented with a crippling disability with ODI assessment, while 59.5% presented with high pain intensity with VAS assessment. MRI assessment of anatomical grading lateral stenosis of L4/L5 level revealed that 45.5% of the patients had grade 2 lateral recess stenosis, 63.6% had grade 2 foraminal stenosis, and 44.6% had extraforaminal stenosis. L5/S1 level analysis showed that 43.0% had grade 2 lateral recess stenosis, 62.0% had grade 2 foraminal stenosis, and 29.8% had extraforaminal stenosis. 64.5% of patients had grade 4 disc degeneration of L4/L5 with mean posterior disc height of 7.0mm ±1.7mm while 59.5% had grade 4 disc degeneration of L5/S1 with mean posterior disc height of 6.3mm ±1.8mm. However, no statistically significant association between clinical symptoms and MRI findings was found.

Conclusions

There was no significant association between the clinical symptoms of pain and disability and the MRI findings for the anatomical gradation of lateral spinal stenosis, the magnitude of posterior disc height, and the extent of disc degeneration. A comprehensive clinical evaluation remains essential for an accurate diagnosis, emphasizing the necessity of appropriately correlating MRI findings with their clinical significance.

## Introduction

Lateral lumbar spinal canal stenosis is a degenerative disease resulting from the cumulative narrowing of the lateral recess and intervertebral foramen of the spinal canal, causing impingement on the nerve root. This narrowing occurs due to the hypertrophy of surrounding osseocartilaginous and ligamentous structures as part of the degenerative process. The anatomical elements of lateral lumbar stenosis can be categorized into lateral recess, foraminal, and extraforaminal stenosis. Degenerative spinal stenosis is a common presentation that can result in significant disability and have a negative impact on the patient's quality of life. Many patients present in their sixth decade of life, where degeneration plays a significant role, and most exhibit lateral canal stenosis affecting both sides of the L4/L5 and L5/S1 levels [1,2].

In clinical practice, magnetic resonance imaging (MRI) is considered the gold standard modality for diagnosing patients with lumbar spinal stenosis [3,4]. Accurate pre-intervention diagnosis is vital to achieving satisfactory treatment outcomes for patients [5].

Previous studies on lumbar stenosis have predominantly focused on patients with central canal stenosis [2], leaving a gap in clinical data and literature concerning the association between clinical symptoms and disability and the severity of lumbar spinal stenosis as determined by MRI. It was anticipated that lateral lumbar spinal stenosis would exhibit more pronounced clinical manifestations due to the limited anatomical space for nerve roots in comparison to central stenosis. However, the diagnosis and assessment of lateral stenosis are often overlooked or underestimated, as there is a lack of a well-defined association between the radiological degree of stenosis and the severity of pain and daily disability.

The primary objective of this study is to assess the connection between clinical symptoms and disability and the anatomical gradation of lateral spinal stenosis, the magnitude of posterior disc height, and the extent of disc degeneration as determined through MRI assessment.

## Materials and methods

This research has been approved by the Human Research Ethics Committee of the authors' affiliated institution with the approval Code USM/JEPeM/17080369, and the patients provided written informed consent.

Patients

This was a cross-sectional study carried out at the University of Sciences, Malaysia, from February 2018 to December 2019. The study subjects involved 121 patients aged 50 years and older who presented at the clinic with clinical presentations suggestive of lateral lumbar spinal stenosis. They underwent magnetic resonance imaging after assessments by orthopedic spine surgeons following established treatment failure over three months of non-operative therapy.

The exclusion criteria for patient selection included those who presented with only back pain, had a primary diagnosis of malignancy, experienced a recent spinal fracture within three months, underwent lumbosacral spinal surgery, had spondylitis, or had congenital spinal anomalies. Patients with cognitive impairment prohibiting completion of the questionnaires were also excluded from the study. By MRI, only patients with moderate central canal stenosis with predominant radiculopathy and claudication clinically were included, excluding patients with severe central canal stenosis.

Assessment of clinical symptoms

The level of disability experienced by the patients was evaluated using the Oswestry Disability Index (ODI) to validate a response to chronic lower back pain, as established by Fritz et al. [6]. It is considered the most effective for the evaluation of persistent severe disability, as concluded by Davies et al. [7]. Most authors use the ODI to evaluate the association and correlation of the disability index with magnetic resonance imaging findings [8-12].

The overall current low back and leg pain severity can be evaluated by a self-administered Visual Analog Scale (VAS) with a range of 0-100mm during outpatient clinic follow-up, as validated by Delgado et al. [13]. Most authors use the VAS to determine the association and correlation of pain intensity with magnetic resonance imaging findings [2,11,12].

Completion of a data collection sheet for demographic data and assessment of clinical symptoms by ODI and VAS questionnaires must be done within three months after the MRI evaluation.

Magnetic resonance imaging

All patients were undergoing the same study protocol for study purposes. MR imaging of the lumbar spine was performed in a supine position with both knees flexed using a 3.0-T MRI system (Achieva 3.0T TX; Philips Healthcare, Best, Netherlands). Fast spin echo (FSE) T1-weighted and T2-weighted images were obtained in the axial and sagittal planes. The protocol comprised sagittal T1 FSE (T1 fast spin echo, TR 400msec, TE 10ms); sagittal T2 FSE (TR 3160msec, TE 120msec); axial T2 FSE (TR 4740msec, TE 120 msec). For all sequences, a 4 mm slice thickness was used. The intersection gap was 0.6-1.3 mm, and the echo train lengths were 6 and 30 for T1 and T2 weighted imaging, respectively.

Imaging examinations

MRI analysis was conducted with the assistance and guidance of a radiologist, involving the qualitative grading of nerve root compression in the lateral recess, foraminal, and extraforaminal areas. Additionally, it included the quantitative grading of posterior disc heights and the qualitative grading of disc degeneration at the bilateral L4/L5 and L5/S1 levels. The analysis was performed in a blinded manner, without knowledge of the clinical findings and radiological reports.

**Figure 1:**
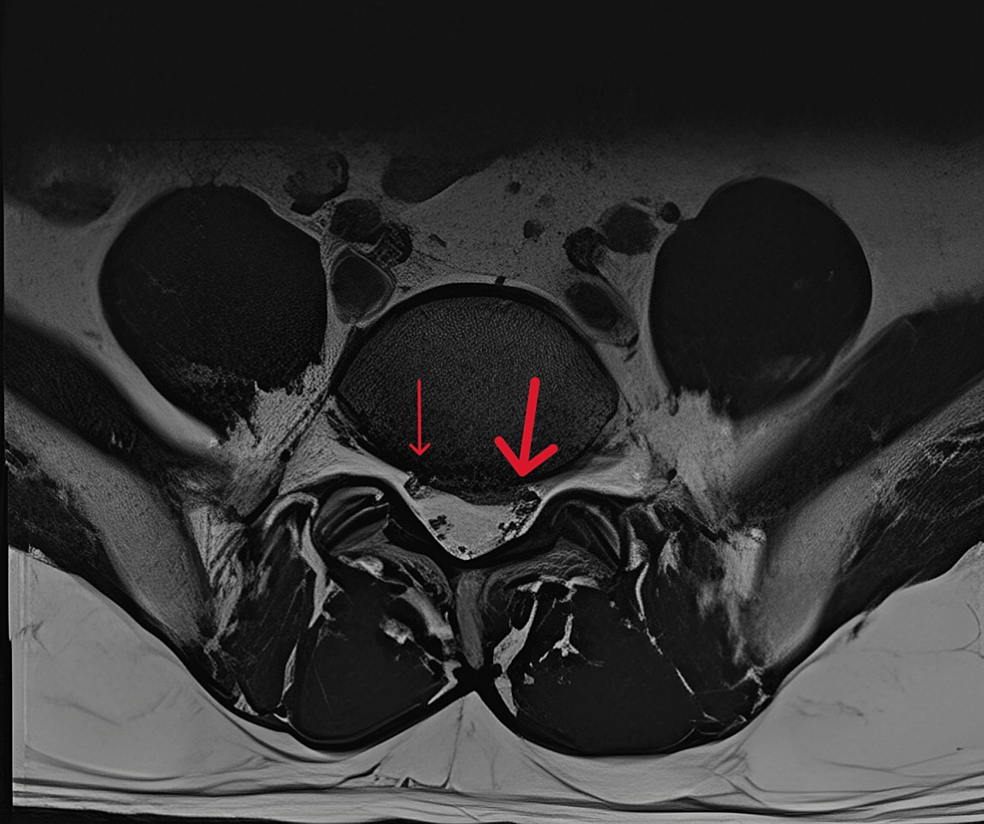
Axial view of T2-weighted MRI, with a small red arrow indicating grade 1 stenosis where the disc is in contact with the nerve root without deviation, while a large red arrow shows grade 2 stenosis with evident deviation of the nerve root

Weishaupt et al. introduced a grading system for nerve root compression in the lateral recess, utilizing T2-weighted images at the axial inferior endplate. The grades were assigned as follows: 0 for no contact of the nerve root with the disc, 1 for nerve root contact without deviation, 2 for nerve root contact with deviation, and 3 for nerve root compression [14]. An illustrative example of MRI evaluation for lateral recess stenosis is depicted in Figure 1, with a small red arrow indicating grade 1 stenosis (disc in contact with nerve root without deviation) and a large red arrow indicating grade 2 stenosis (evident deviation of the nerve root).

**Figure 2:**
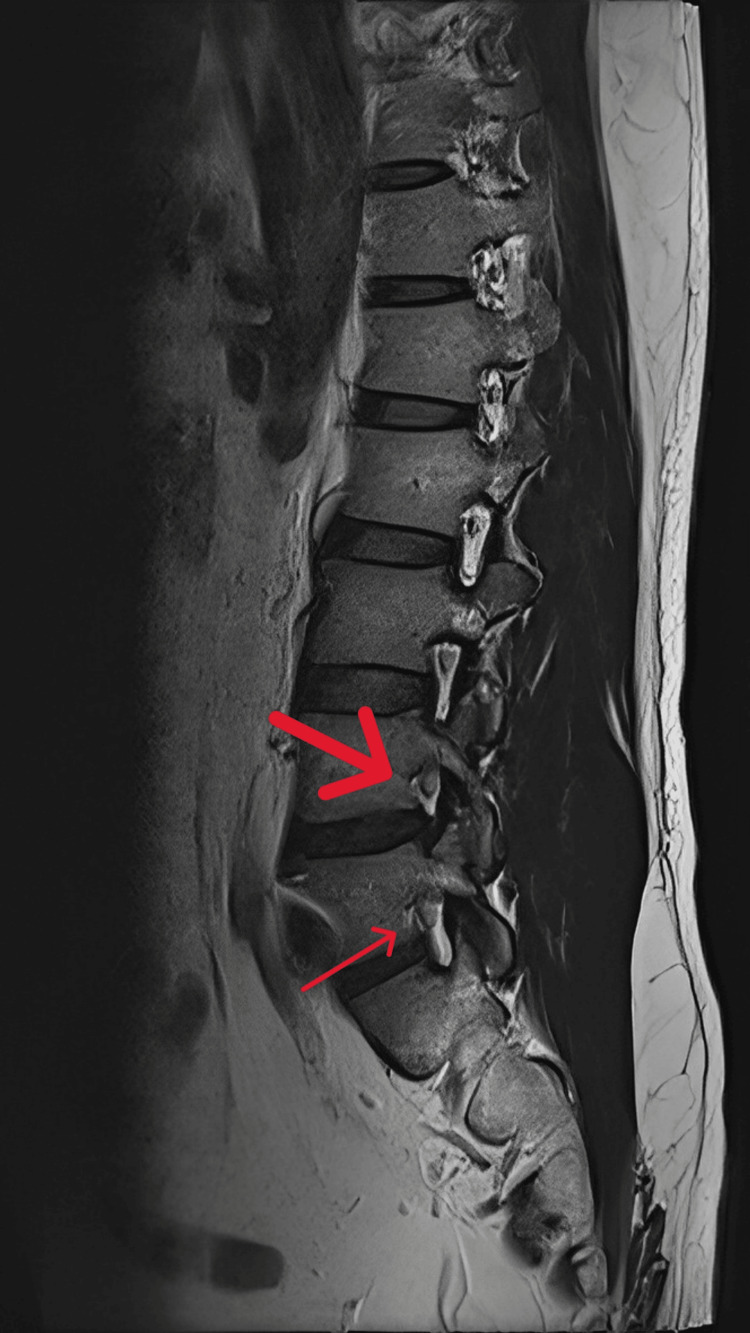
Parasagittal view of T1-weighted images of MRI, with a small red arrow indicating grade 1 stenosis, featuring compression of the epidural fat where the remaining fat still completely surrounds the nerve root, and a large red arrow indicating grade 2 stenosis, where the epidural fat only partly surrounds the nerve root

The qualitative assessment of foraminal stenosis, based on T1-weighted parasagittal images, was graded as follows: grade 0 for normal foramina, grade 1 for mild foraminal stenosis, grade 2 for moderate foraminal stenosis, and grade 3 for severe stenosis. This grading was determined by Wildermuth et al. [15]. An example of MRI evaluation for foraminal stenosis is shown in Figure 2, with a small red arrow indicating grade 1 stenosis and a large red arrow indicating grade 2 stenosis (epidural fat only partly surrounding the nerve root).

**Figure 3:**
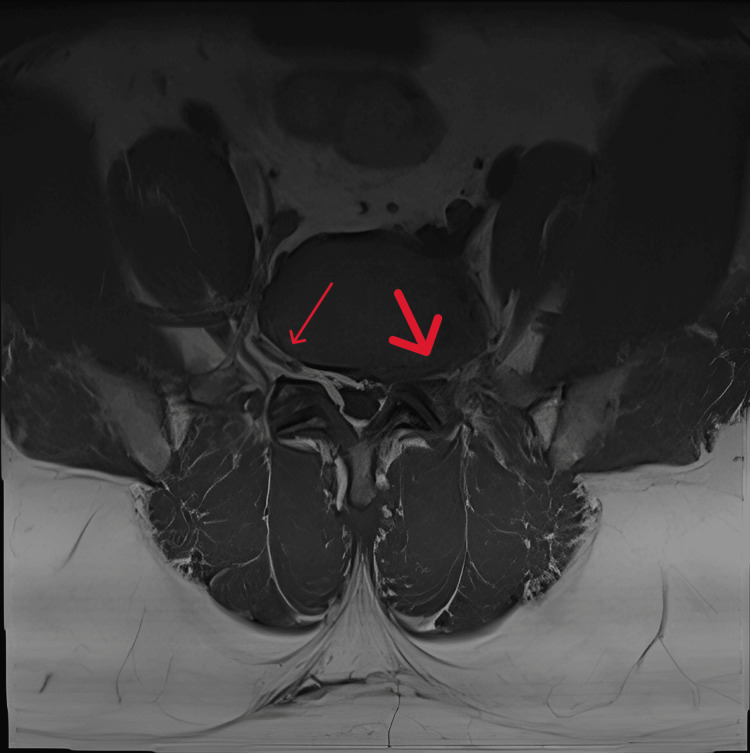
Axial view of T1-weighted MRI images, with a small red arrow indicating no stenosis and a large red arrow indicating extraforaminal stenosis with an absence of perineural fat signal

Extraforaminal nerve root entrapment was evaluated from T1-weighted axial images at the center of a disc, with an evident circumferential loss of perineural fat signal, and was graded as either yes or no entrapment [16-18]. The MRI assessment of extraforaminal stenosis is depicted in Figure 3, with a small red arrow indicating no stenosis and a large red arrow indicating extraforaminal stenosis (absence of perineural fat signal).

Cinotti et al. proposed a quantitative assessment of posterior disc height, calculated from a T2-weighted mid-sagittal view as the shortest distance between the adjacent superior and inferior endplates [19]. Pfirrmann's Grading System was used to score the degree of lumbar intervertebral disc degeneration from a T2-weighted midsagittal view. The grades were as follows: 1 for a homogeneous bright white disc with a clear distinction of nucleus and annulus structure, 2 for an inhomogeneous bright white disc with a clear distinction of nucleus and annulus structure, 3 for an inhomogeneous grey disc with an unclear distinction of nucleus and annulus structure and a slightly decreased disc height, 4 for an inhomogeneous grey to black disc with a loss of distinction of the nucleus and annulus structure and a moderately decreased disc height, and 5 for an inhomogeneous black disc with a loss of distinction of the nucleus and annulus structure and a collapsed disc space [20,21].

Statistical analysis

Data analysis was conducted using Statistical Packages for Social Science (SPSS) version 23.0 and STATA version 14.0. When patients had multilevel spinal stenosis, the level and side with the worst stenosis were selected for the study's association. The same principle was applied for the assessment of posterior disc height and grade of disc degeneration in L4/L5 and L5/S1, with the worst score chosen for the association analysis.

The correlation between posterior disc height and ODI and VAS scores was evaluated using Pearson's correlation test via SPSS version 23.0 (IBM Inc., Armonk, New York).

The association between the extent of lateral stenosis and disc degeneration on MRI with ODI and VAS scores was determined by the Fisher Exact Test via STATA version 14.0 (StataCorp LLC, College Station, Texas). This test was chosen as a replacement for the Chi-squared test, as it is more accurate for small cell sizes with expected values less than five. The result of the association was considered statistically significant, with a p-value of less than 0.05.

## Results

A total of 121 patients were clinically evaluated for degenerative lateral lumbar spinal stenosis, and patient characteristics are summarized in Table 1. The assessment of ODI scores showed that patient symptoms and disability ranged from a minimal score of 24% to a maximal score of 92%, with a mean value of 62.2% ± 10.7%. Based on the percentage disability score of the ODI, out of the 121 patients, one patient (0.8%) demonstrated moderate disability, 53 patients (43.8%) had a severe disability, 60 patients (49.6%) were crippled, and seven patients (5.8%) were bedridden.

**Table 1:** Clinical characteristics of the study subjects with lateral spinal stenosis (n=121) Data are numbers of patients (n), with percentages in parentheses or means, ± standard deviations in parentheses ODI - Oswestry Disability Index; VAS - Visual Analogue Pain Scale

Variables	Results
Age (year)	58.7 (range 50 -77)
Male/female	57/64 (47.1/52.9)
Married	118 (97.5)
Height (cm)	158.2 (12.0)
Weight (kg)	71.9 (11.5)
BMI (kg/m2)	29.1 (6.0)
ODI score	62.2 (10.7)
VAS score	79.3 (8.6)

According to VAS scores, patient pain intensity ranged from a minimal score of 55 to a maximal score of 90, with a mean value of 79.3 ± 8.6. In the overall VAS scores, six patients (5.6%) had severe pain (scores 41-60), 72 (59.5%) had high pain (scores 61-80), and 43 (35.5%) had very high pain (scores 81-100). None of the patients had minimal to moderate pain. Table 2 summarizes the analysis of anatomical lateral stenosis at L4/L5, while Table 3 provides the analysis for L5/S1 based on MRI findings.

**Table 2:** Number of grades of lateral lumbar stenosis for lumbar levels L4/L5 (n=121) Data are numbers of patients (n), with percentages in parentheses

L4/L5 level	Grades			
	0	1	2	3
Lateral Recess n (%)	7 (5.8)	18 (14.9)	55 (45.5)	41 (33.9)
Intraforaminal n (%)	0	27 (22.3)	77 (63.6)	17 (14.0)
Extraforaminal n (%)	54 (44.6)			

**Table 3:** Number of grades of lateral lumbar stenosis for lumbar levels L5/S1 (n=121) Data are numbers of patients (n), with percentages in parentheses

L5/S1 level	Grades			
	0	1	2	3
Lateral Recess n (%)	7 (5.8)	50 (31.4)	52 (43.0)	12 (9.9)
Intraforaminal n (%)	2 (1.7)	25 (20.7)	75 (62.0)	19 (15.7)
Extraforaminal n (%)	36 (29.8)			

Through MRI analysis, the posterior disc height at L4/L5 demonstrated a mean of 7.0 mm ± 1.7 mm, ranging from 2.3 mm to 11.9 mm. Similarly, at the L5/S1 level, the mean posterior disc height was 6.3 mm ± 1.8 mm, ranging from 1.5 mm to 10.4 mm. The evaluation of intervertebral disc degeneration at L4/L5 and L5/S1 is detailed in Table 4.

**Table 4:** Grades of lumbar disc degeneration for lumbar levels L4/5 and L5/S1 (n=121) Data are numbers of patients (n), with percentages in parentheses

Lumbar level	Grades				
	1	2	3	4	5
L4/L5 n (%)	0	0	36 (29.8)	78 (64.5)	7 (5.8)
L5/S1 n (%)	0	1 (0.8)	34 (28.1)	72 (59.5)	14 (11.6)

**Figure 4:**
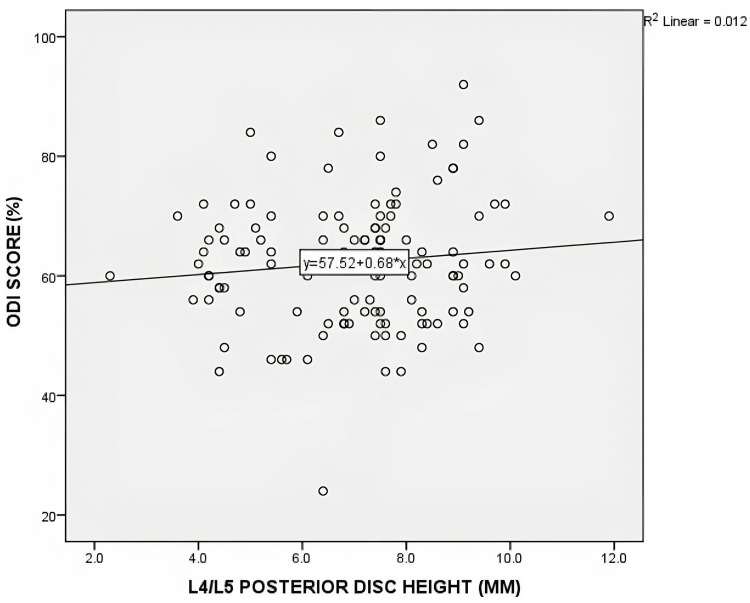
Scatterplot graph of correlation between the posterior disc height of L4/L5 on MRI with ODI score (n=121) MRI - magnetic resonance imaging; ODI - Oswestry Disability Index scale

**Figure 5:**
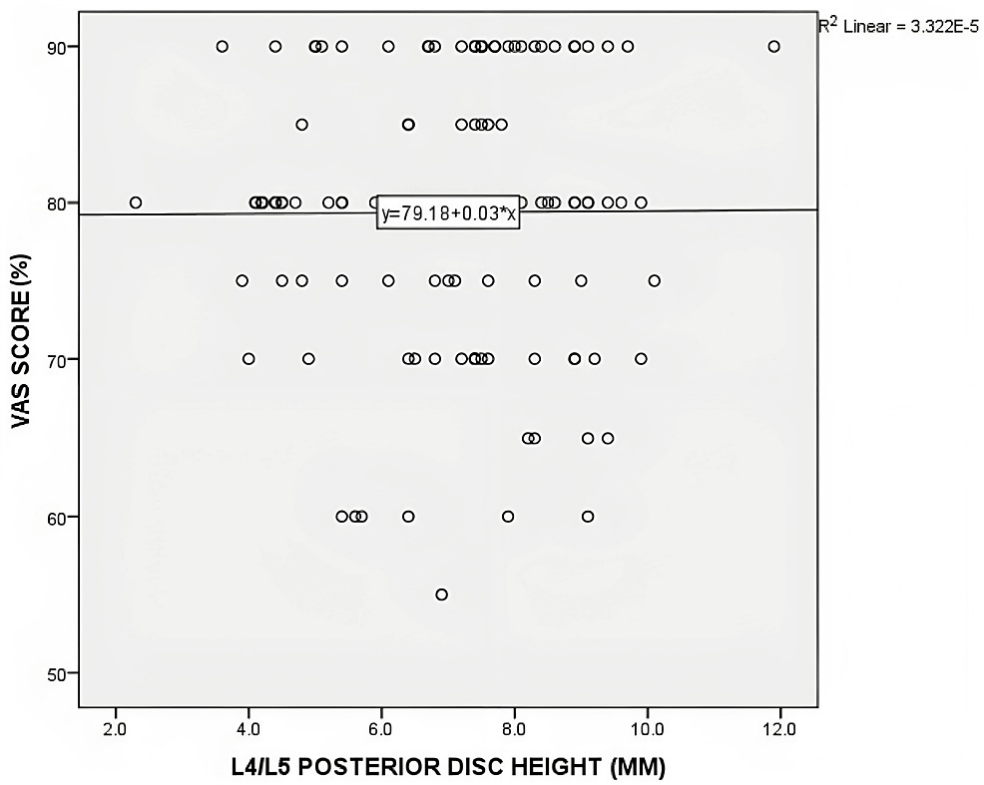
Scatterplot graph of correlation between the posterior disc height of L4/L5 on MRI with VAS score (n=121) MRI - magnetic resonance imaging; ODI - Oswestry Disability Index scale

**Figure 6:**
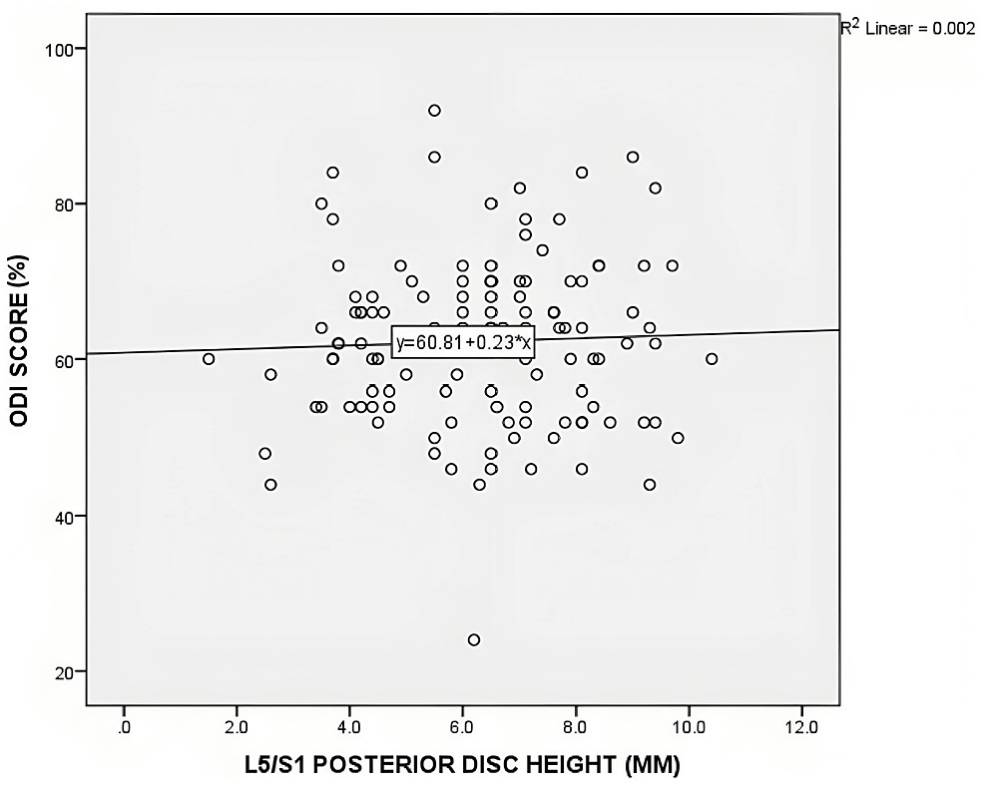
Scatterplot graph of correlation between the posterior disc height of L5/S1 on MRI with ODI score (n=121) MRI - magnetic resonance imaging; ODI - Oswestry Disability Index scale

**Figure 7:**
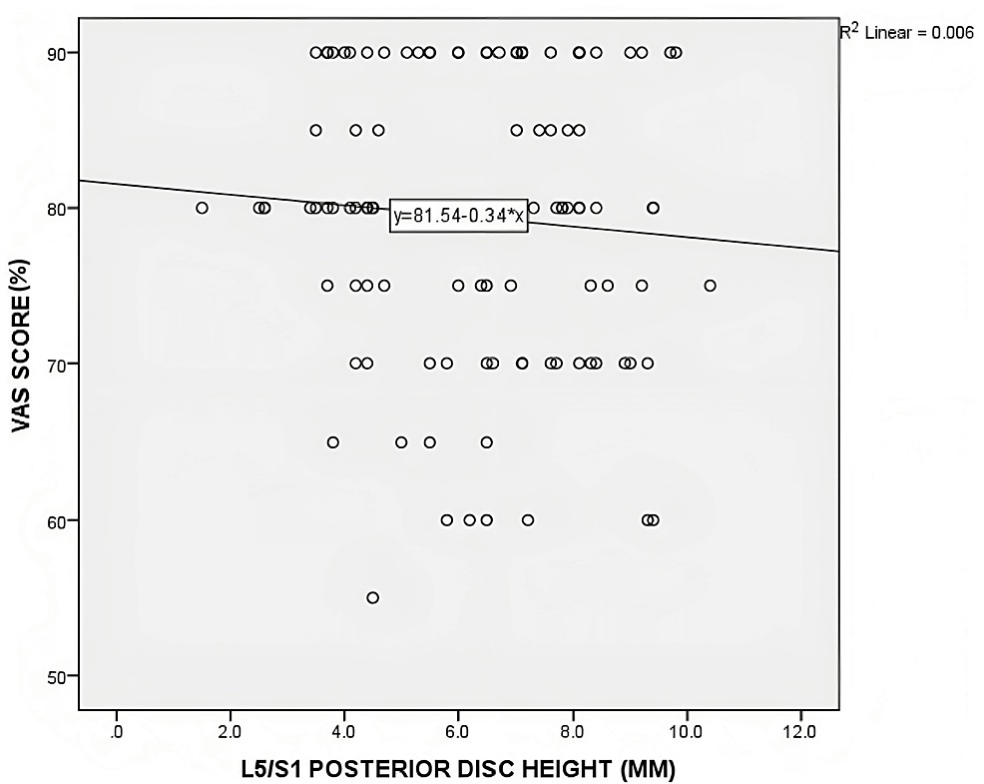
Scatterplot graph of correlation between the posterior disc height of L5/S1 on MRI with VAS score (n=121) MRI - magnetic resonance imaging; ODI - Oswestry Disability Index scale

No statistically significant correlation was found between posterior disc height and the distribution of ODI and VAS scores. For the L4/L5 level, the Pearson's correlation coefficient (r) was 0.11 (p=0.22) for ODI and 0.06 (p=0.95) for VAS. At the L5/S1 level, the correlation coefficients were -0.41 (p=0.65) for ODI and 0.74 (p=0.41) for VAS. Figures 4-7 depict scatterplots illustrating the relationship between posterior disc height and the distribution of ODI and VAS scores.

Further statistical analysis, conducted in line with the study's objectives, revealed no statistically significant association between the distribution of ODI and VAS grading, the anatomical grading of lateral recess stenosis, foraminal stenosis, extraforaminal stenosis, or disc degenerative grading, as detailed in Table 5.

**Table 5:** Association between the anatomical grading of lateral recess stenosis, anatomical grading of foraminal stenosis, grading of the extraforaminal stenosis and the disc degenerative grading based on MRI with ODI and VAS grading (n=121) Data are number of p-value based on the Fisher-Eexact test* All p-values less than 0.05 were considered, as statistically significant. MRI - magnetic resonance imaging; ODI - Oswestry Disability Index scale

MRI findings	ODI*	VAS*
Grading of lateral recess stenosis	0.824	0.822
Grading of foraminal stenosis	0.622	0.227
Grading of the extraforaminal stenosis	0.442	0.952
Disc degenerative grading	0.562	0.966

## Discussion

Based on our findings, the mean age of the sample participants was 58.7 years. We specifically targeted the sixth decade of life to yield optimal results, as this period significantly contributes to the degenerative process, particularly due to relative estrogen deficiency, resulting in a higher prevalence among females.

The majority of the patients were married or cohabiting, which is important to achieve good results since almost all the patients have similar family environmental and psychosocial contributions. This similarity is reflected fairly in the severity of symptoms, as it was the main determinant for the subjective experience of pain and disability among the patients. Most of the patients had an abnormal body mass index, with 76% of them being overweight or obese. This finding is consistent with the fact that obesity is strongly related to biomechanical changes contributing to degenerative lumbar stenosis. An increased body mass index will result in higher shear forces that overload the joints and torque on the lumbar disc, potentially leading to facets and disc degeneration.

Analysis of lateral lumbar stenosis revealed a notably higher prevalence of lateral recess stenosis at the L4/L5 level, affecting 79.4% of patients with moderate to severe compression, compared to 52.9% at the L5/S1 level. The prevalence of moderate to severe foraminal stenosis was consistent at both levels, affecting 77.7% of the patients. These findings align with previous studies indicating a greater occurrence of severe lateral stenosis at the L4/L5 level and severe foraminal stenosis at the L5/S1 level based on MRI assessments and their association with clinical symptoms in the general population, as concluded by Ishimoto et al. [22]. This observation can be explained by the susceptibility of the lower lumbar region, especially the L4/L5 level, to high mechanical stress, connecting a mobile segment of the lumbar spine to a relatively rigid sacrum and pelvis.

Examining the prevalence of extraforaminal stenosis, the L4/L5 level showed a higher involvement (44.6%) compared to the L5/S1 level (29.8%). This result is consistent with a prior study by Lee et al. that reported a 39.5% occurrence of extraforaminal stenosis [23]. The phenomenon is attributed to the loss of intervertebral disc height due to disc degeneration, leading to the anterosuperior subluxation of the superior articular process of the inferior vertebra, causing stenosis.

The calculated mean posterior disc height in symptomatic patients was 7.0 ± 1.7mm for the L4/L5 level and 6.3±1.8mm for the L5/S1 level, significantly lower than the measurements in normal subjects, which were 10.1±1.0mm and 8.5 ± 1.0mm, respectively [24]. A separate cadaveric dissection study by Cinotti et al. showed an average posterior disc height of 6.55 ± 1.7mm for L4/L5 and 5.29 ± 1.9mm for L5/S1 [19].

The majority of our patients exhibited degenerated lumbar discs, with 64.5% graded as Pfirrmann grade 4 for L4/L5 and 59.5% for L5/S1. Additionally, 5.8% were graded as 5 for L4/L5, and 11.6% for L5/S1. In comparison, a previous study reported lower rates of disc degeneration, with Pfirrmann grade 4 at 34.5% for L4/L5 and 33.7% for L5/S1, while showing similar findings for L5/S1 disc degeneration as published by Middendorp et al. [9]. The observed differences are likely due to degenerative changes within the intervertebral discs, characterized by the loss of water content, diminished nutritional transport, and reduced proteoglycan content. Disc aging leads to changes, particularly in the nucleus, becoming less gelatinous and more fibrous. These significant changes can manifest as the loss of homogeneous brightness of the disc with a diminished clear distinction between the nucleus and annulus, as well as a decrease and collapse of the disc height as observed on MRI."

Our study suggests challenges in reliably diagnosing lateral lumbar stenosis based solely on imaging findings, as there appears to be an inconsistency between clinical symptoms and imaging results. This inconsistency may be attributed to the limited capability of MRI in identifying nerve root compression adequately.

Static images of canal dimensions might not predict a patient's symptoms without assessing the dynamic nature of the disease process. The degree of compression is dynamic and likely varies based on the patient's condition. The limitations of our study lie in conducting routine clinical MRI with patients in a supine position, which may not reflect symptoms that worsen in an upright position due to alterations in nerve element compression. Therefore, upright MRI imaging, especially under axial loading, becomes crucial for a comprehensive assessment, as it causes displacement of anatomical structures leading to nerve root compression, not observed in the supine position, as suggested by Beattie et al. [25].

The absence of association in our study might also be linked to the fluctuating nature of symptoms over time, potentially following a natural course that could either improve or remain stable, thereby affecting the perceived pain and disability of the patient [26].

Pain and disability experienced by the patient are subjective and influenced by emotional, psychological, and genetic factors. Although we evaluated disability using the ODI score, which is widely accepted and has strong psychometric properties, it remains subjective and may not consistently correlate with the severity of radiological spinal stenosis [27].

A comprehensive history and thorough physical examination are essential for diagnosing degenerative lateral spinal stenosis. While MRI evidence of nerve compression is necessary, it should be clinically assessed before being attributed solely as the cause of back pain. Therapy should be directed towards the patient's most disturbing symptoms rather than solely relying on the severity of radiographic narrowing.

This study aims to establish a robust predictive value concerning the relationship between clinical symptoms, disability, and MRI imaging. The research featured a selectively chosen elderly population aged 50 years and above, specifically targeting those with typical presentations while excluding patients with severe central stenosis. To ensure data quality, symptom recording and disability assessment were carried out solely by the principal investigator, while a detailed visual qualitative MRI analysis was conducted by an experienced radiologist.

Several limitations were identified in this study. It's important to note that this was a cross-sectional study, limiting its ability to provide conclusive evidence for the broader population. Furthermore, the study encompassed a relatively small sample size recruited exclusively from our center. Additionally, MRI evaluations were not conducted at the peak of clinical symptoms and disability but rather within three months of their presentation due to constraints in immediate MRI availability.

## Conclusions

In summary, our findings conclude that there is no significant interrelation between clinical symptoms, pain severity, the extent of daily disability, and the observed MRI results for the anatomical gradation of lateral spinal stenosis, the magnitude of posterior disc height, and the extent of disc degeneration. Lumbar spinal stenosis remains a clinical-radiological syndrome, and a comprehensive clinical evaluation remains essential for an accurate diagnosis, emphasizing the necessity of appropriately correlating MRI findings with their clinical significance. On the other hand, MRI is a gold standard diagnostic tool for decision-making in the management and intervention of patients with spinal stenosis.

## Figures and Tables

**Figure 8:**
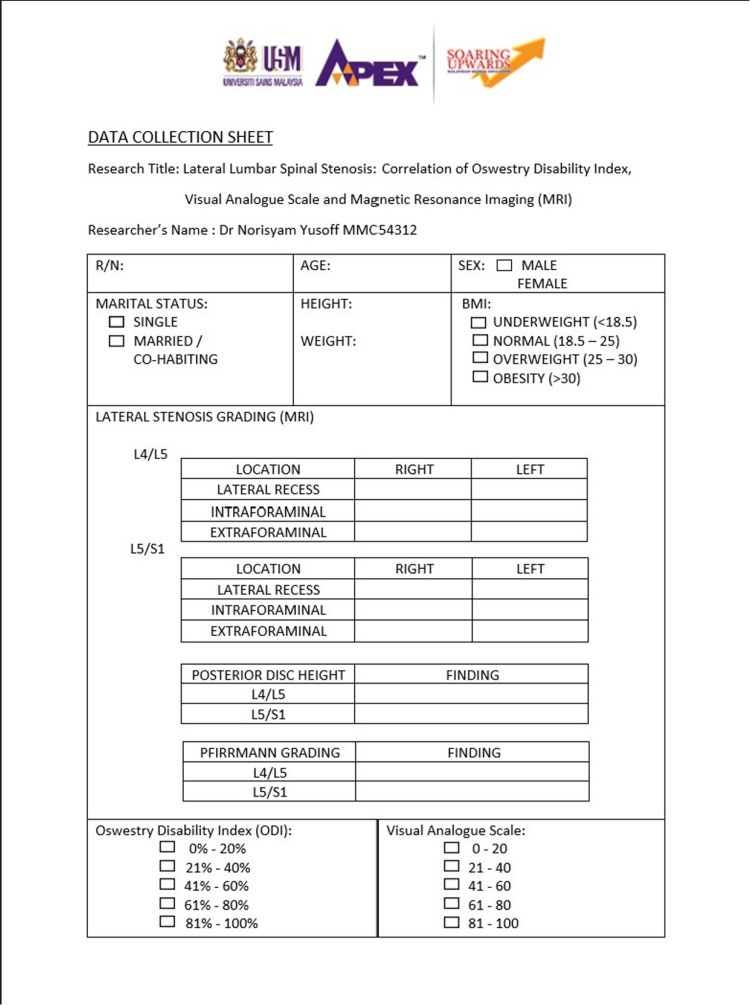
Data collection sheet.

**Figure 9:**
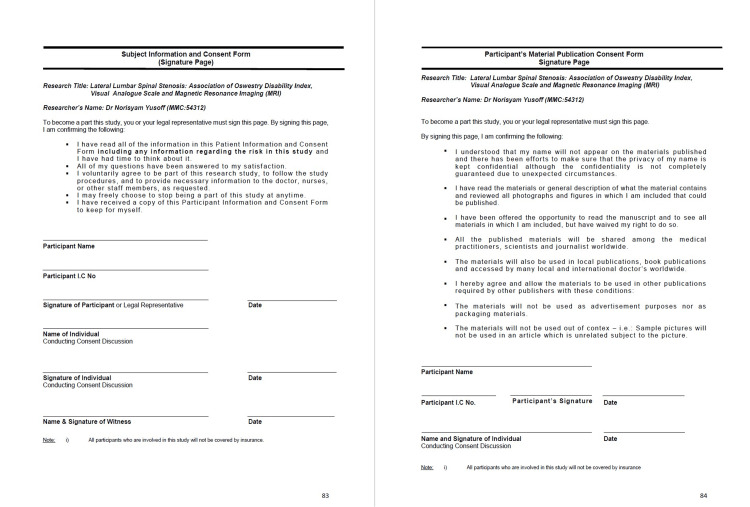
Subject information and consent form with participant's material publication consent form.

**Figure 10:**
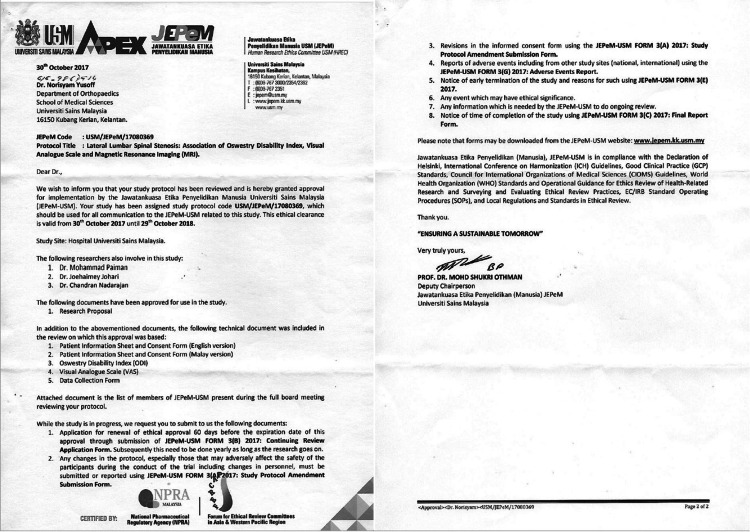
Human Research Ethics Committee approval letter.

## Appendices

Below are the supplemental materials for the study, including the data collection sheet (Figure 9), the subject information and consent form, which incorporates the participant's material publication consent form (Figure 10), and the Human Research Ethics Committee approval letter (Figure 11).
